# Divalent metal cofactors differentially modulate RadA-mediated strand invasion and exchange in *Saccharolobus solfataricus*

**DOI:** 10.1042/BSR20221807

**Published:** 2023-02-23

**Authors:** Corey J. Knadler, William J. Graham V, Michael L. Rolfsmeier, Cynthia A. Haseltine

**Affiliations:** School of Molecular Biosciences, Washington State University, Pullman, Washington 99164-7520, U.S.A.

**Keywords:** archaea, ATPase, RadA recombinase, RecA family recombinases, Saccharolobus solfataricus, Walker box mutants

## Abstract

Central to the universal process of recombination, RecA family proteins form nucleoprotein filaments to catalyze production of heteroduplex DNA between substrate ssDNAs and template dsDNAs. ATP binding assists the filament in assuming the necessary conformation for forming heteroduplex DNA, but hydrolysis is not required. ATP hydrolysis has two identified roles which are not universally conserved: promotion of filament dissociation and enhancing flexibility of the filament. In this work, we examine ATP utilization of the RecA family recombinase SsoRadA from *Saccharolobus solfataricus* to determine its function in recombinase-mediated heteroduplex DNA formation. Wild-type SsoRadA protein and two ATPase mutant proteins were evaluated for the effects of three divalent metal cofactors. We found that unlike other archaeal RadA proteins, SsoRadA-mediated strand exchange is not enhanced by Ca^2+^. Instead, the *S. solfataricus* recombinase can utilize Mn^2+^ to stimulate strand invasion and reduce ADP-binding stability. Additionally, reduction of SsoRadA ATPase activity by Walker Box mutation or cofactor alteration resulted in a loss of large, complete strand exchange products. Depletion of ADP was found to improve initial strand invasion but also led to a similar loss of large strand exchange events. Our results indicate that overall, SsoRadA is distinct in its use of divalent cofactors but its activity with Mn^2+^ shows similarity to human RAD51 protein with Ca^2+^.

## Introduction

Homologous recombination (HR) functions as a potentially error-free method for the repair of DNA double-strand breaks and plays a fundamental role in cell survival and evolution [1–4]. At the center of all HR pathways are the RecA family recombinases (RecA in bacteria, Rad51 in eukaryotes, and RadA in archaea) which catalyze the strand invasion, the homology search, and the formation of heteroduplex DNA [5–7]. The process by which these recombinases perform these actions results from the contributions of several separate activities. Each of these recombinases are capable of ATP-dependent ssDNA binding and form structurally dynamic helical nucleoprotein filaments [8–18]. These nucleoprotein filaments extend ssDNA into a conformation that is competent for homology comparison with dsDNA. RecA filaments randomly bind dsDNA and scan small regions for homology before dissociating if matches are not found [18–21]. For both *Escherichia coli* RecA and *Saccharomyces cerevisiae* Rad51, homology recognition involves short triplet-based sequences of at least nine bases; with *E. coli* RecA, this sampling is expanded to larger triplet-based steps as strand exchange progresses [19,22,23].

While ATPase activity does not appear to be necessary for these processes, the ability to hydrolyze ATP is conserved in all RecA family recombinases identified to date and they all include Walker box ATPase motifs [6,24–29]. The ATPase activities of both *E. coli* RecA and *S. cerevisiae* Rad51 have been shown to improve the selectivity of the homology search during invasion and strand exchange [22,23,30]. Additionally, *E. coli* RecA ATPase activity prevents the accumulation of toxic, non-productive RecA complex formation [30] and hydrolysis-dependent filament cohesion has been observed, where non-contiguous filaments shift by 1–2 nucleotides to be ultimately positioned in phase with each other [31]. Although ATPase activity is unnecessary for strand exchange, it has been shown to promote the process [32–35]. ATPase mediated disassociation from DNA has been reported for *E. coli* RecA, human RAD51, *S. cerevisiae* Rad51, and *S. solfataricus* RadA [18,36–39].

Prevention of dissociation, or increased recombinase-DNA association, is improved by the actions of additional factors that inhibit ATPase activity. For example, recombinase ATPase activity is reduced while DNA association is improved by *S. solfataricus* Ral1 or SsoRadA Walker box mutants, use of ATP analogs with *E. coli* RecA or *S. cerevisiae* Rad51, or replacing Mg^2+^ with Ca^2+^ as the divalent cofactor for human RAD51 [20,34,36–38,40]. Still other influences can improve ATPase activity by enhancing DNA binding, such as the presence of low concentrations of DinI protein or use of ATPγS with *E. coli* RecA [41,42]. The complex relationship between DNA binding and recombinase ATPase activity is the result of requisite DNA binding for ATPase activity, while ATPase inhibition reduces dissociation kinetics, ultimately improving binding. Recombinase protein ATPase activity has been universally found to act as a conformational switch that changes both the rigidity and physical geometry of the nucleoprotein filament [18,33,36,43]. The ATP-bound state of *E. coli* RecA, human RAD51, *S. cerevisiae* Rad51, and *S. solfataricus* RadA stretches ssDNA, forming an ordered conformation capable of performing strand exchange [8,9,18,36,44–46]. Hydrolysis of ATP to ADP, or direct binding of ADP, causes *E. coli* RecA and human RAD51 to form shorter, more flexible filaments [18,44–46]. At present, it is unclear whether the mechanistic role played by ATP-induced conformational changes is shared among recombinases.

In addition to ATP binding and hydrolysis, recombinase activities are also dependent on divalent cations. Historically, the biochemical focus has been on Mg^2+^, but further investigation of recombinases has revealed that other cations such as Ca^2+^, function to modulate their activity [25,33,34,47,48]. Ca^2+^ has been shown to enhance *Methanococcus voltae* and *Methanococcus maripaludis* RadA as well as human RAD51-mediated heteroduplex DNA formation and to inhibit their ATPase activities [25,33,34]. Thus far, the role of alternative cofactors in recombinase protein activity modulation for members of the crenarchaea has been unclear. In this work, we examine the effect of three divalent cations, Mg^2+^, Ca^2+^, and Mn^2+^, on ATPase activity, strand invasion, and strand exchange mediated by the *S. solfataricus* RadA recombinase protein.

## Materials and methods

### Protein purification

SsoRadA, the K120A and K120R ATPase mutants, and *Thermotoga maritima* pyruvate kinase (*Tm*PK) and *Thermotoga maritima* lactate dehydrogenase (*Tm*LDH) were from laboratory stocks and purified as previously described [38,49]. Except where otherwise noted, SsoRadA was purified in the presence of 1 mM EDTA.

### ATPase assays

ssDNA-dependent ATPase activity for SsoRadA purified in the presence or absence of EDTA was determined using the thermophilic continuous coupled spectrophotometric assay essentially as previously described [50]. All assays were performed at 80°C with a Beckman Coulter DU-800 spectrophotometer fitted with a Peltier device to maintain temperature. Conversion of NADH (Sigma) to NAD was measured at 340 nm. Reactions were performed in a 150 μl volume and contained 40 mM HEPES (pH 7.0), 20 mM MgOAc, 300 μM NADH, 2 mM phospohenol pyruvate, 15 U *Tm*LDH, 15 U *Tm*PK, 7 μM nucleotide φX174 ssDNA (NEB), 0.5 mM ATP, and used ultrapure deionized water with a resistance of 18.2 megohms. Mixtures were equilibrated at 80°C for 3 min prior to assay initiation by addition of 3 μM SsoRadA. All assays were performed a minimum of three times. The extent of ATP hydrolysis was determined by measuring NADH oxidation and its extinction coefficient of 6220 M^−1^cm^−1^. Maximal velocities were determined by the 5-min window with the highest change of NADH concentration and averaging ATPase reaction velocity across all points of all replicates during that window. Maximal velocities are reported +/- the standard deviation of velocities within this window.

All other ATPase activity determinations were through use of the thin layer chromatography (TLC) assay essentially as previously described [51] with the following alterations. Reaction mixtures contained 25 mM Tris acetate (pH 7.5), 100 μg/ml BSA, 0.5 mM ATP, 5-10 μCi [γ−^32^P] ATP, 15 μM SsoRadA, 50 μM (nucleotides) ϕX174 virion (NEB), ultrapure deionized water with a resistance of 18.2 megohms, and the indicated cation at 10 mM concentration. Assays were incubated at 65°C for 15 minutes and samples were then applied to PEI cellulose sheets (Analtech) that were pre-run with ddH_2_O. Plates were developed in 3.8% formic acid and 0.25 M LiCl and allowed to air dry. The amount of ATP hydrolyzed was imaged and analyzed by phosphorimaging using a Storm 850 Phosphorimager (Molecular Dynamics). The Image Quant TL program was used for quantitation and included a minimum of three replicates.

Kinetic parameters for SsoRadA with MgCl_2_ were determined by initial velocity measurement of reactions containing 20 μM (nucleotides) ϕX174 virion (NEB) and 0.5 μM SsoRadA. Additionally, a range of ATP from 0.5 to 150 μM ATP was used, depending on the divalent metal cofactor and time of incubation was from 0.5-120 minutes to maintain measurable activity. Reaction yields in μM were divided by incubation duration to calculate initial reaction velocity. Initial rates were calculated by dividing the reaction velocity by SsoRadA protein concentration (μM). These initial rates were then graphed as a function of initial ATP concentration, with *k*_cat_ and *K_m_* calculated using a non-linear regression of (eqn 1).

(1) $$rate=\frac{kcat*[substrate]}{Km+[substrate]}$$

*k*_cat_ was calculated as the rate at the horizontal asymptote and *K*_m_ was set to be the midpoint between 0 and the horizontal asymptote. To determine IC50s for Mg^2+^ and Mn^2+^ with respect to ADP and ATPγS inhibition the reaction rate across a range of ATPγS or ADP concentrations was measured and graphed as a non-linear regression of inhibitor concentration versus response (GraphPad 9) [52].

### Filter binding assays

Filter binding was used to determine *K*_D_ values for protein-ATP and protein-ssDNA binding essentially as previously described [38] ATP binding reactions were performed in the absence of ssDNA and contained 40 mM HEPES (pH 7.0), 10 mM CaCl_2_, MgCl_2_, or MnCl_2_, 50 mM KCl, 20 pmol of SsoRadA, ATP at varying concentrations, and used ultrapure deionized water with a resistance of 18.2 megohms. Reactions to assess ssDNA binding included 3 μM ATP and varying concentrations of the 63-mer oligonucleotide 5′-ACAGCACCAATGAAATCTATTAAGCTCCTCATCGTCCGCAAAAATATCGTCACCTCAAAAGGA-3′ (Sigma Aldrich). Binding curves were determined with a minimum of three replicates where *K*_D_ values as reported in the text were generated using Prism 9 (GraphPad) and error represents the standard deviation.

### Displacement loop assay

Assay substrates were pBR322 plasmid prepared without alkali lysis as previously described [49] and the homologous 101-mer oligonucleotide DLpBR322 5′-TGGCCTGCAACGCGGGCATCCCGATGCCGCCGGAAGCGAGAAGAATCATAATGGGGAAGGCCATCCAGCCTCGCGTCGCGAACGCCAGCAAGACGTAGCCC-3′ synthesized by Sigma-Aldrich and purified by HPLC. DLpBR322 was 5′ end labeled with [γ-^32^P] using 10 U of T4 polynucleotide kinase (NEB) and its manufacturer supplied buffer. Labeling was for 1 h at 37°C followed by enzyme heat inactivation at 75°C for 10 min. Assay reactions contained 25 mM Tris acetate (pH 7.5), 10 μg/ml BSA, 0.9 μM nucleotides DLpBR322, 2 mM ATP, and 0.6 μM SsoRadA, K120A, or K120R protein as indicated in the text, and used ultrapure deionized water with a resistance of 18.2 megohms. Samples containing the ATP regeneration system included 10 U of *Tm*PK and 2 mM of phosphoenolpyruvate (Alfa Aesar). Reactions were incubated at 65°C for 5 min after which pBR322 was added to a final concentration of 30 μM base pairs and incubation was continued for the times indicated. Assays were stopped by the addition of EDTA and SDS to final concentrations of 25 mM and 1.5% respectively and 0.4 U Proteinase K (NEB), followed by incubation at 65°C for 30 min. About 4 μl of loading buffer containing 0.025% bromophenol blue in 30% glycerol was added to each reaction before loading on a vertical 1.5% agarose, 1× TAE (40 mM Tris acetate and 2 mM EDTA) gel. Electrophoresis was for 150 min at 75 V. Gels were dried and exposed to a Molecular Dynamics phosphorimaging screen and results were visualized using a Storm 850 phosphorimager. Images were analyzed using Image Quant TL software and results were background adjusted and quantitated relative to the 1-min timepoint for wild-type SsoRadA with CaCl_2_. Product formation for all other conditions were compared to this value and expressed as fold change. All assays were performed with a minimum of three replicates.

### DNA strand exchange reactions

SsoRadA at an 11 μM concentration was incubated with ϕX174 ssDNA (NEB) at a concentration of 33 μM nucleotides in 30 mM MES (pH 6.5), 5 μg/ml BSA, 2.5 mM ATP, and 10 mM of divalent cation as indicated in ultrapure deionized water with a resistance of 18.2 megohms for 5 min at 80°C. Reactions were initiated by the addition of 33 μM nucleotides *Pst*I linearized ϕX174 RF1. Following addition of dsDNA, reactions were further incubated at 65°C for the times indicated. Samples that included ATPγS contained the specified amount of the ATP analog in addition to 2.5 mM ATP while those that included the ATP regeneration system also had 2 mM phosphoenolpyruvate and 3.13 units of *Tm*PK. Assays were stopped by deproteinization by addition of EDTA and SDS to final concentrations of 25 mM and 1.5% respectively and 0.4 U Proteinase K (NEB), followed by incubation at 65°C for 20 min. Reaction products were separated by electrophoresis for 16 h at 30 V on a 1%, 1× TAE (40 mM Tris acetate and 2 mM EDTA) agarose gel. Following electrophoresis, gels were washed with 50 mM EDTA for 1 h then with ddH_2_O three times for 20 min each time. The gels were then stained with 0.5 μg/ml ethidium bromide for 1 h before being photographed using a Syngene GeneFlash system and quantified with Licor Image Lite software. Utilization of the dsDNA substrate and production of nicked circular dsDNA and joint molecule intermediate products were quantified by determining relative band intensities using raw volumes and adjusting for background values and reported as percent yield.

### Graphing and non-linear regression

All graphs were generated using GraphPad Prism 9 and all error bars represent ±standard deviation.

## Results

### SsoRadA exhibits differential ATPase activity when purified in the absence of EDTA

**Figure 1:**
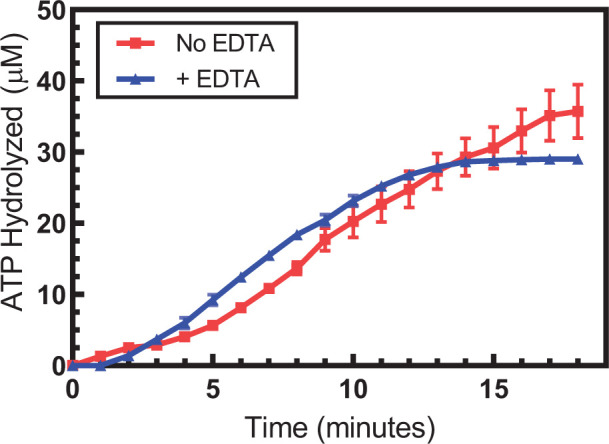
Protein purification in the presence or absence of EDTA impacts SsoRadA ATPase activity ATPase activity of SsoRadA purified in the presence (blue triangles) or absence (red squares) of EDTA was measured using the coupled assay across 1-min timepoints. Reactions included 3 μM SsoRadA and 7 μM ssDNA (nucleotides). Each graphed point represents four replicates and error bars are standard deviation.

In previous work from our lab and others [6,49–51,53], heterologously expressed SsoRadA protein has been purified using buffers containing the chelating agent EDTA, which removes divalent metal cofactors. Controlled concentrations of divalent cofactor, historically Mg^2+^, are then provided in the assay buffer for activity determinations. The effect of removal of the cofactor and/or substitution with another, has not been established for SsoRadA. To investigate the role of divalent cofactors with SsoRadA, we purified the protein in the absence of EDTA and compared ATPase activity with protein purified with the chelator (Figure 1). When EDTA is excluded from the purification protocol, we found that both initiation and diminution of ATPase activity is slowed (red squares), but there is no apparent alteration in maximal hydrolysis velocity, which was observed as 2.93 μM*min^−1^ ± 1.40 and 2.94 μM*min^−1^ ± 0.49 for SsoRadA purified without EDTA and with EDTA respectively. Previous reports for SsoRadA as well as other RecA family recombinases including *E. coli* RecA and *S. cerevisiae* Rad51 show no ATP hydrolysis activity in the absence of Mg^2+^, indicating that free ATP is unable to act as a substrate and the presence of a divalent cation is necessary for function [6,7,54]. The observation of differential SsoRadA ATPase activity in Figure 1 raises the question as to whether a divalent metal cofactor (other than Mg^2+^) remains associated with SsoRadA following heterologous expression that is involved in its function.

### Mn^2+^ and Ca^2+^ reduce SsoRadA ATPase activity while only Ca^2+^changes ATP binding affinity

**Figure 2:**
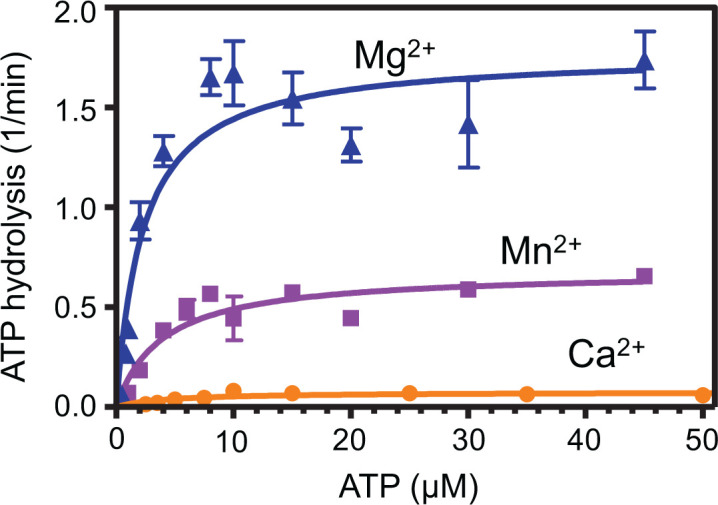
Different divalent cofactors alter SsoRadA ATPase activity Kinetic parameters of SsoRadA ATPase activity were determined by TLC and a range of ATP concentrations in the presence of 20 μM ssDNA and 500 nM protein. Cofactors were added at a concentration of 10 mM where blue triangles are Mg^2+^, orange circles are Ca^2+^, and purple squares are Mn^2+^. Each graphed point represents a minimum of five replicates and lines are the non-linear regression of each dataset using (eqn 1) (experimental procedures). Error bars are standard deviation.

Divalent cations such as Mg^2+^ are commonly used by recombinases as cofactors for binding and hydrolysis of ATP and substitution of Mg^2+^ for Ca^2+^ reduces the ATPase activity of *E. coli* RecA, *Methanococcus voltae* RadA, and human RAD51, [20,25,34,47]. Cation dependence has not been broadly examined for members of the crenarchaea, so it is unclear whether Ca^2+^ mediated enhancement is a general feature of archaeal recombinases or is instead specific for methanogen recombinases. To further investigate the role of divalent cations for SsoRadA function, we tested the effect of Ca^2+^ and Mn^2+^ on ATPase activity. Cation concentrations were selected based on SsoRadA activity in the highly sensitive strand exchange reaction [51] and titration experiments. At 10 mM cation concentration, we did not observe strand exchange product limitation and elected to use that amount for cation impact evaluations. Kinetic parameters were first determined in the presence of an excess of DNA. As shown in Figure 2, there is a dramatic effect upon substituting for Mg^2+^. The observed *K*_cat_ with Mg^2+^ was 1.7 min^−1^ ± 0.09, Mn^2+^ was 0.68 min^−1^ ± 0.04 (2.5-fold lower than with Mg^2+^), and Ca^2+^ was 0.075 min^−1^ ± 0.005 (23-fold lower than with Mg^2+^). Relative to Mg^2+^, the observed *K*_m_ was quite similar for each cofactor with 2.3 μM ± 0.47 for Mg^2+^, 3.9 μM ± 0.84 for Mn^2+^, and 4.3 μM ± 1.2 for Ca^2+^. We additionally measured the *K*_D_ of SsoRadA-ATP binding in the presence of each cofactor using a filter binding assay. Observed *K_D_* values for SsoRadA-ATP binding were 1.6 μM ± 0.44 and 1.13 μM ± 0.44 for Mn^2+^ and Mg^2+^, respectively (±standard deviation), indicating that these cofactors have a minor effect on ATP binding. The *K*_D_ for SsoRadA-ATP binding with Ca^2+^ was above 800 μM, and therefore too high to be accurately determined. Taken together, the substantial difference between the observed *K*_m_ and *K*_D_ for SsoRadA in the presence of Ca^2+^ suggests that this cofactor destabilizes the association between SsoRadA and ATP.

### Ca^2+^ and Mn^2+^ both enhance SsoRadA-mediated strand invasion

**Figure 3:**
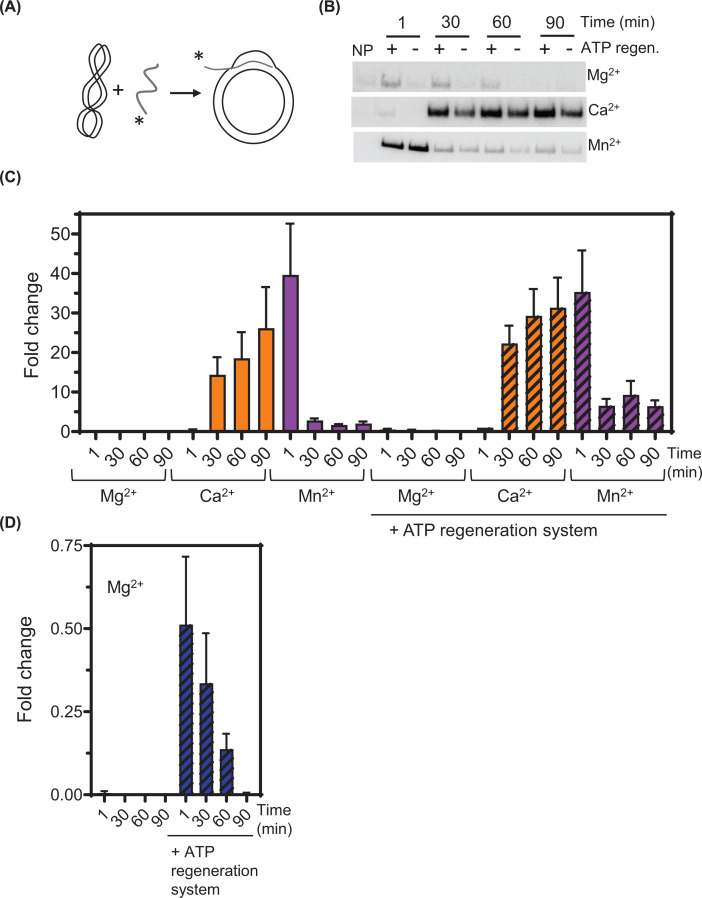
D-loop formation mediated by WT SsoRadA in the presence of different divalent cations (**A**) Schematic of the D-loop reaction. (**B**) Representative autoradiographs showing the final invasion product for reactions in the presence of Mg^2+^, Ca^2+^, or Mn^2+^, and with or without an ATP regeneration system. (**C** and **D**) Quantitation of D-loop formation time courses. Reactions including the ATP regeneration system are indicated along the *x*-axis. Results are expressed as fold increase relative to WT SsoRadA with Ca^2+^ and ATP regeneration at the 1-min time point. Results shown in (**D**) are identical to those for WT SsoRadA data (**C**) in the presence of Mg^2+^, but with reduced *y*-axis compression to show fold change. For panels (C and D) bars with crosshatch patterning represent reactions that include the ATP regeneration system. All data are the averages of at least three reactions and error bars are ± standard deviation.

To further understand the impact of cations on SsoRadA function, we next investigated recombinase-mediated strand invasion using a displacement or D-loop assay. In this assay, the ability of the recombinase to form a nucleoprotein filament on ssDNA that can invade a homologous double-stranded circular plasmid is measured and invasion product yield and kinetics can be determined. We tested SsoRadA invasion activity with each of the divalent cations both with and without the inclusion of an ATP regeneration system, which maintains a high ATP:ADP ratio. Relative to Mg^2+^ in the absence of the regeneration system, inclusion of either Ca^2+^ or Mn^2+^ significantly increases strand invasion yield (Figure 3, compare C and D). In the case of Ca^2+^, this increase is apparent after 30 min of incubation and is close to 25-fold after 90 min. For Mn^2+^, there is an initial strong increase in invasion up to about 40-fold which is highly reduced as incubation continues. The characteristic D-loop cycle [38,55] is apparent for SsoRadA with both Mn^2+^ and Mg^2+^ where an initial burst of product formation is followed by a reduction at later time-points (Figure 3C,D, purple and blue bars). When Ca^2+^ was used, however, maximum yield was achieved at the latest time point and no significant dissociation was apparent (Figure 3C, orange bars). These results suggest that the increased yield of invasion products in the presence of Mn^2+^ is likely the result of increased strand invasion activity while increased yield in the presence of Ca^2+^ could be due to D-loop dissociation failure, increased strand invasion activity, and/or persistence of strand invasion activity over time. When the ATP regeneration system was included, substantial enhancement of SsoRadA-mediated strand invasion was observed with Mg^2+^ (Figure 3D) indicating that the ATP-bound state is necessary for strand invasion. This result is consistent with previous reports for *E. coli* RecA, human Rad51, and archaeal RadA recombinases [56–58]. For reactions containing either Ca^2+^ or Mn^2+^ there was a smaller effect, suggesting that these cofactors reduce either SsoRadA affinity for ADP, changing the stability of the ADP-bound SsoRadA-ssDNA nucleoprotein filament, or alternatively permit SsoRadA to initiate strand invasion while ADP is bound (Figure 3C).

### SsoRadA Walker box ATPase mutant strand invasion activity is differentially altered

**Figure 4:**
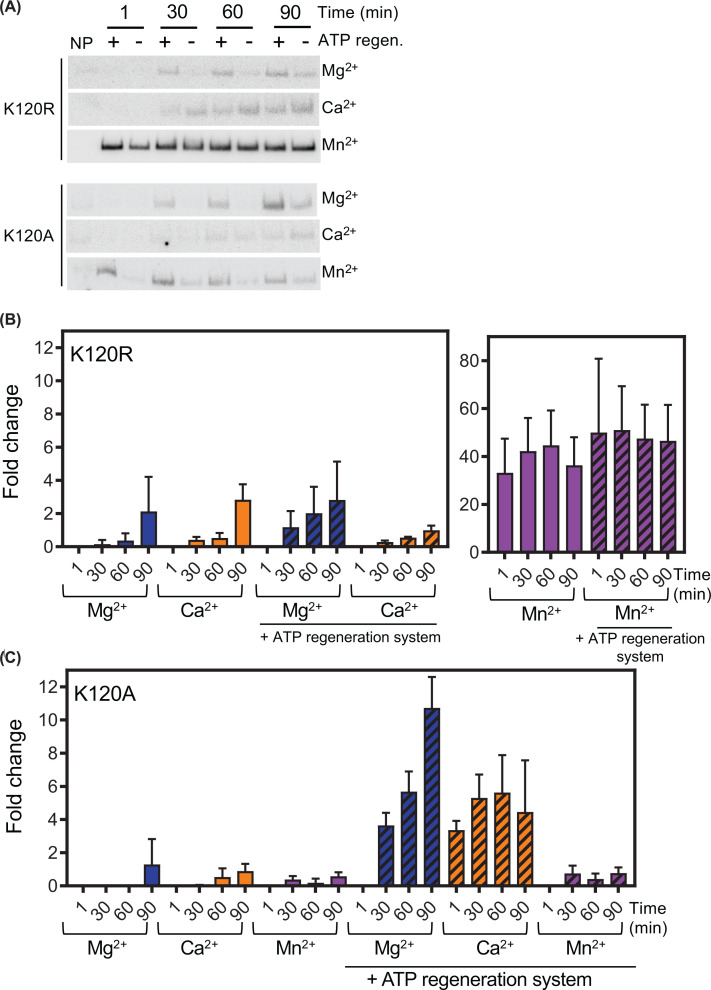
RadA ATPase mutants display altered responses to different cofactors (**A**) Representative autoradiographs of D-loop time courses. Divalent cations tested are indicated, as is the presence/absence of the ATP regeneration system. Invasion products for each condition were measured at 1-, 30-, 60-, and 90-min time points. (**B**) D-loop formation quantitation for the K120R mutant protein. Mn^2+^ data are shown in a separate panel from Mg^2+^ and Ca^2+^ to highlight variations. (**C**) D-loop formation quantitation for the K120A mutant protein. For all data points shown in panels (B and C), a minimum of three replicates were used. Results are expressed as fold increase relative to WT SsoRadA with Ca^2+^ and ATP regeneration at the 1-minute time point. For panels (B and C) bars with crosshatch patterning are reactions that include the ATP regeneration system. Error bars represent standard deviation in all cases.

As there was an apparent effect on SsoRadA invasion activity dependent on high ATP:ADP ratio, we next examined mutations in the Walker A ATPase domain. The Walker A domain is one of two conserved motifs that are components of the nucleotide-binding site of ATP hydrolyzing proteins [59]. The lysine residue in the GKT/S of the A box contacts the γ-phosphate of ATP and is essential for binding [60]. We previously constructed two Walker A ATPase mutants for SsoRadA [38]. In these mutants, the conserved lysine residue located at position 120 has been altered to either an alanine (K120A) or an arginine (K120R). Examining strand invasion activity for these altered proteins in this work, we determined that when Mg^2+^ is used as a cofactor, both mutants can catalyze strand invasion. The K120A protein-mediated strand invasion products appeared slowly relative to those of the K120R mutant. Changing the divalent cofactor from Mg^2+^ to either Mn^2+^ or Ca^2+^ resulted in a clear alteration in strand invasion activities. As shown in Figure 4, the K120R mutant with Mn^2+^ displayed the highest yield, producing invasion products at a level close to 20-fold higher than with either Mg^2+^ or Ca^2+^ (panel B, compare solid purple bars to blue and orange bars). D-loop invasion products in the presence of Mn^2+^ were formed rapidly and no significant changes of invasion product levels were observed over time. Conversely, use of either Mg^2+^ or Ca^2+^ resulted in invasion product accumulation at later time points. Inclusion of the ATP regeneration system had little effect on maximal invasion product yield in any case except at the 90-min time point with Ca^2+^, where levels were reduced relative to those produced at a higher ATP:ADP ratio (Figure 4B, compare orange solid bars to cross-hatched bars). With the K120A mutant, use of Mg^2+^ resulted in invasion product levels comparable to those seen with K120R (Figure 4C). When the ATP regeneration system was included, however, maximal D-loop formation was increased by approximately 10-fold (Figure 4C, compare blue solid bars to cross-hatched bars). An increased ATP:ADP ratio resulted in a modest increase invasion product formation by the K120A mutant when Mn^2+^ was present (Figure 4C, purple cross-hatched bars). Reactions containing Ca^2+^ and the ATP regeneration system showed a higher overall increase in invasion relative to those without higher levels of ATP (Figure 4C, compare orange solid bars to cross-hatched bars).

Interestingly, the slow formation of strand invasion products and apparent absence of net dissociation of D-loops observed in several of the reactions with ATPase mutant protein reflects the activity of wild-type SsoRadA in the presence of Ca^2+^. In this case, D-loops also form slowly without measurable dissociation. This result is contrary to observed invasion product disassociation with wild-type SsoRadA with either Mg^2+^ or Mn^2+^. D-loop disassociation with these cofactors could indicate that SsoRadA protein fails to remain strand invasion competent after completion of the reaction, as has been previously reported for RecA protein [55]. The lack of apparent invasion product dissociation with wild-type RadA in the presence of Ca^2+^ and by both mutants with all cations tested suggests that strand invasion activity is maintained at later time points in the reaction or that D-loops have become more stable.

### Ca^2+^ and Mn^2+^ reduce SsoRadA-mediated strand exchange

**Figure 5:**
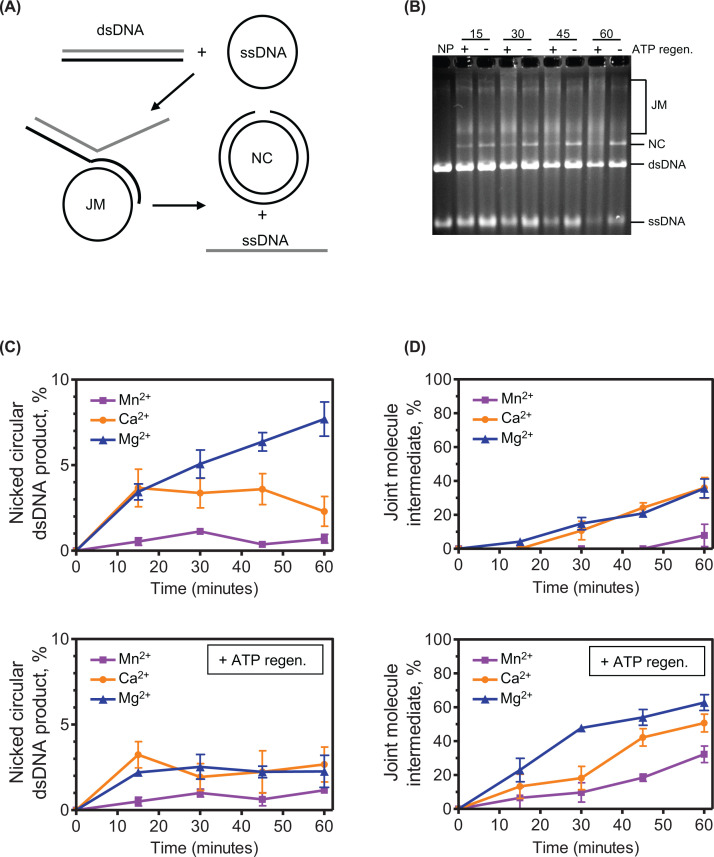
Ca^2+^ and Mn^2+^ reduce SsoRadA-mediated strand exchange (**A**) Schematic of the strand exchange reaction with input ss-DNA, dsDNA, intermediate joint molecule (JM), and final nicked circular (NC) products illustrated. (**B**) Representative gel indicating the presence or absence and duration of reactions above the gel for assays performed with Mg^2+^ as the cation. NP indicates a reaction where no protein was added. The panels in (**C**) show the yield of JM product as a percentage of total DNA and NC product as a percentage of total DNA in the panels in (**D**). For all reactions, SsoRadA was used at a stoichiometry of 1:3 (nucleotides). Blue triangles indicate the use of Mg^2+^, orange circles show Ca^2+^, and Mn^2+^ is represented with purple squares. All graphed data points are the averages of a minimum of three replicates and error bars represent ± standard error.

To determine the impact of alternate divalent cations on the strand exchange function, we used an established *in vitro* assay with plasmid-length homologous substrates [51,61]. With this assay both strand-invaded intermediates (joint molecules) and fully exchanged products (nicked circular) can be directly visualized on an agarose gel (Figure 5, panels A and B). The highest yield of nicked circular product was observed in reactions containing Mg^2+^, with an approximately 50% reduction in yield with Ca^2+^; activity with Mn^2+^ was less than 20% of that with Mg^2+^ (Figure 5C, top panel). Additionally, use of Mg^2+^ resulted in accumulation of nicked circular DNA over time while Ca^2+^ led to cessation of product formation after the first 15 minutes (Figure 5C, top panel, compare orange circles to blue triangles). When either Mg^2+^ or Ca^2+^ were provided, joint molecule yields produced by SsoRadA were similar but substituting Mn^2+^ resulted in fewer joint molecules and delayed intermediate formation (Figure 5D, top panel). The overall abrogation of joint molecule formation and strand exchange with Mn^2+^ was unexpected, since Mn^2+^ had only a mild effect on SsoRadA ATPase activity and enhanced recombinase-mediated strand invasion (Figures 2 and 3C, compare purple to blue). This result suggests that SsoRadA-produced heteroduplex DNAs in short, D-loop level sizes could have different cofactor or stability requirements than those necessary for generation of larger, multi-kilobase strand exchange products.

We also tested the effect of increasing levels of ATP available during strand exchange by including the regeneration system and with the three divalent cofactors (Figure 5C,D, bottom panels). Joint molecule formation was altered significantly for reactions containing Mg^2+^, which showed earlier formation and higher overall production. Both Ca^2+^ and Mn^2+^ also showed increased joint molecule formation, although levels were not as high as those observed for Mg^2+^. Production of completed nicked circular DNAs, however, was reduced for Ca^2+^ and Mg^2+^ when compared with reactions that did not have the ATP regeneration system. This may indicate that SsoRadA fails to complete the exchange reaction when ATP is in abundance and that the DNA molecules are caught at the strand invasion step. The ATP:ADP ratio may be important for completion of strand invasion with larger substrates, as inclusion of the ATP regeneration system increases the persistence of joint molecules with all the tested cofactors. This suggests that completion of the exchange process could be more efficient with the ADP-bound state of SsoRadA.

### Altering the ATP:ADP ratio depresses K120A mutant strand invasion and exchange with Mg^2+^

**Figure 6:**
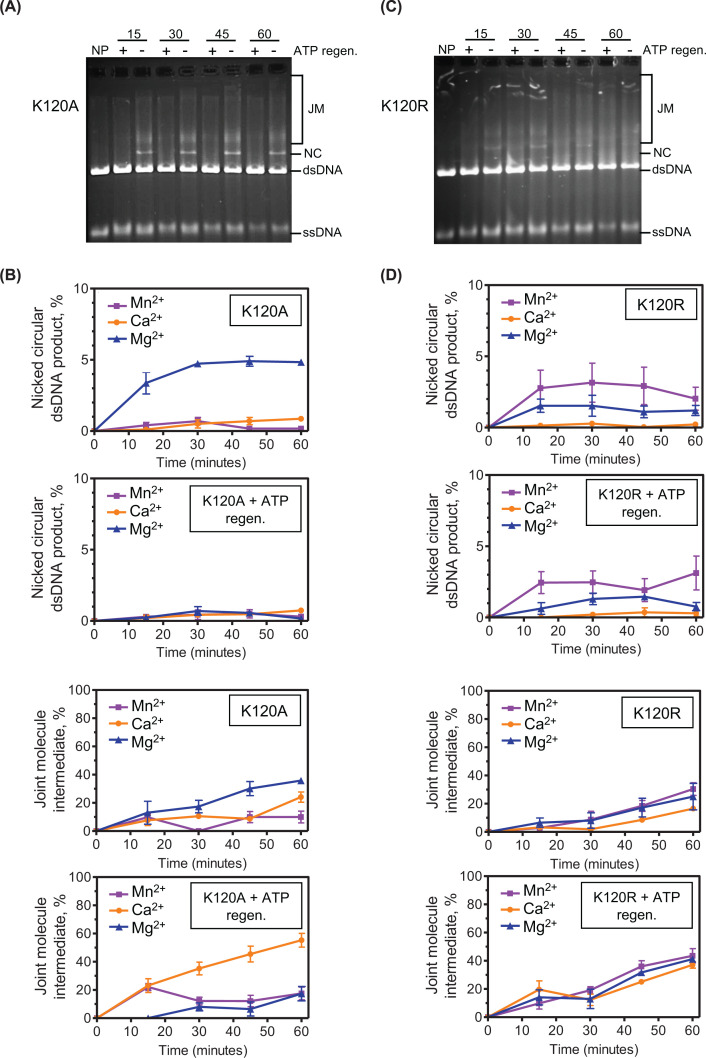
The K120A mutant is more sensitive to ATP:ADP ratios than the K120R mutant in strand exchange Representative gels of strand exchange assays containing the K120A and K120R mutants are presented in panels (**A** and **C**). Quantification of NC products in panels (**A** and **C**) is shown in the bottom panels of (**B** and **D**), respectively. Quantification of JM products from panels (**A** and **C**) is shown in the top panels (B and D), respectively. Reactions containing the ATP regeneration system are indicated on the corresponding graphs. Reactions with Mg^2+^ are represented with blue triangles, Ca^2+^ are orange circles, and Mn^2+^ are purple squares. All points are averages of at least three reactions and error bars represent ± standard deviation.

As levels of available ATP have an apparent impact on the overall strand exchange process, we turned to the Walker A box K120A and K120R SsoRadA ATPase mutants [38] to better understand what role binding and hydrolysis play in the mechanism. Both mutant proteins show significant differences in strand invasion and exchange activity relative to each other and the wild-type protein. Total yield of nicked circular DNA produced by K120A with Mg^2+^ was approximately two-thirds that generated by wild-type SsoRadA under the same conditions (compare Figure 6B, top panel to Figure 5C, top panel, blue triangles) while joint molecule yields were similar between the two proteins (compare Figure 6B, third panel to Figure 5D top panel, blue triangles). Substituting Mg^2+^ with either Ca^2+^ or Mn^2+^ resulted in an overall reduction of nicked circular DNA product (Figure 6B, top panel). Depletion of ADP (and concurrent increase in ATP) by addition of the ATP regeneration system with Mg^2+^ dramatically reduced production of nicked circular products (Figure 6B, second panel). Joint molecule production relative to reactions lacking the regeneration system was also reduced in the presence of Mg^2+^ but was increased for Ca^2+^ for the timepoints examined (at least 2-fold) and for early Mn^2+^ timepoints and 3-to 4-fold for the later time points examined (Figure 6B, compare third and bottom panels).

For the K120R mutant the highest yield of nicked circular product was observed with Mn^2+^ and was comparable to that produced by wild-type protein with Ca^2+^ and near 50% of that produced by wild-type protein with Mg^2+^ at later time points. (compare Figure 6D top panel with Figure 5C bottom panel). Joint molecule yields for this mutant with either Mg^2+^ or Mn^2+^ in the absence of the ATP regeneration system were similar to those obtained with the wild-type protein with Mg^2+^, with yields near 20–30% of total product at the 60-min timepoint (Figure 6D, third panel and 5D, top panel). This result differs from that seen for the wild-type protein with Mn^2+^, where joint molecule formation is extremely low. Inclusion of the ATP regeneration system increased joint molecule formation by 1.5-fold with Mg^2+^ and 2-fold with Ca^2+^ but had no apparent effect on nicked circular product (Figure 6D).

Taken together, these results suggest that alteration of the SsoRadA Walker A box GKT motif to either GAT or GRT impairs strand exchange but enhances strand invasion activity when the ATP:ADP ratio is high. At the same time, this high ratio with Mg^2+^ as the divalent cofactor has a distinct effect on the K120A mutant, where it impairs joint molecule production and effectively abolishes conversion to nicked circular product. This result is not apparent for the K120R mutant, which is instead largely unaffected by ATP:ADP ratios. Thus, K120A but not K120R may need to bind free ADP to successfully perform strand exchange. This is consistent with the previously reported activities for these mutants, where K120R hydrolyzes ATP more effectively than K120A and may more readily adopt an ADP-bound conformation in the absence of free ADP [38].

### ATPγS inhibits SsoRadA strand exchange in the presence of Mg^2+^ but not Ca^2+^

**Figure 7:**
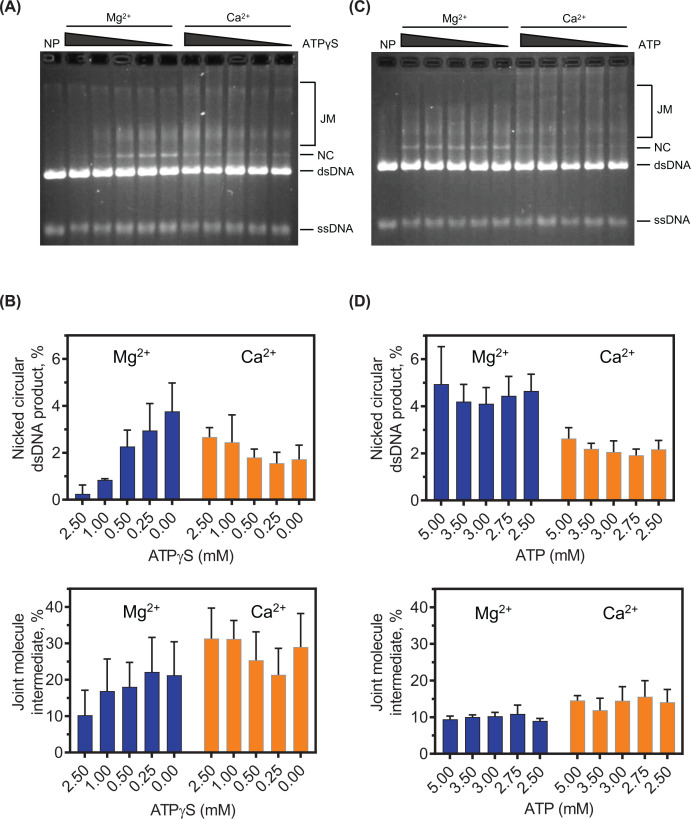
Ca^2+^ protects SsoRadA mediated strand exchange from inhibition by ATPγS Thirty-minute endpoint strand exchange reactions were performed to measure the effect of ATPγS on SsoRadA activity with either Mg^2+^ or Ca^2+^. (**A**) Representative gel for reactions including ATPγS; concentrations are at 2.5, 1, 0.5, 0.25, and 0 mM from left to right. (**B**) Quantification of nicked circular product and joint molecule formation in the presence of ATPγS. (**C**) Representative gel for control reactions performed in the absence of ATPγS. (**D**) Quantification of nicked circular product and joint molecule formation without ATPγS. For all reactions, ATP is at a concentration of 2.5 mM and in cases where ATPγS is omitted, equivalent concentrations of ATP were used in place of ATPγS. For graphs in panels (B and D), blue bars represent Mg^2+^ containing reactions and orange bars represent those with Ca^2+^. All points are averages of a minimum of three replicates and error bars are ± standard deviation.

To further probe ATP hydrolysis requirements during strand exchange, we substituted ATP with the slowly hydrolyzed ATP analog ATPγS. Since Mn^2+^ produced low levels of nicked-circular product (Figure 5C), we focused only on Mg^2+^ and Ca^2+^ for these assays. As shown in Figure 7B (top panel), SsoRadA-mediated strand exchange in the presence of Mg^2+^ is sensitive to inhibition by ATPγS and reaches nearly complete inhibition of nicked circular dsDNA product formation at equimolar amounts (2.5 mM) of ATP and ATPγS. Joint molecule formation was also inhibited by ATPγS, but the effect was much less extreme (Figure 7B, bottom panel). SsoRadA produced less nicked circular dsDNA product with Ca^2+^ and there was no apparent inhibitory effect with ATPγS for either joint molecule formation or completed strand exchange (Figure 7B). To ensure that the presence of additional nucleotide was not responsible for the observed effects, we evaluated SsoRadA strand exchange with ATP concentrations equal to the combined ATP + ATPγS concentrations represented in Figure 7B. As shown in Figure 7D, levels of nicked circular and joint molecule product formation remained constant with increased ATP for both Mg^2+^ and Ca^2+^ containing conditions. Collectively, these results suggest that SsoRadA-mediated strand exchange is dependent on ATP hydrolysis when Mg^2+^ is the divalent cofactor but independent with Ca^2+^. Thus, SsoRadA ATP hydrolysis in the presence of Mg^2+^ may provide the necessary filament dynamics to allow strand exchange to proceed while substitution with Ca^2+^ could lead to increased filament destabilization, reducing the need for hydrolysis.

### Divalent cofactors modulate recombinase ssDNA binding and Mn^2+^ reduces ADP inhibition of ATPase activity

To test whether Ca^2+^ can destabilize the SsoRadA nucleoprotein filament, we determined SsoRadA-ssDNA *K*_D_ values in the presence of all three divalent cofactors with or without ATP using a filter binding assay. *K*_D_ values for Mg^2+^, Mn^2+^, and Ca^2+^ were 21.3 nM ± 1.6, 23.1 nM ± 14.4, and 186.8 nM ± 13.2 (±standard deviation) respectively in the presence of ATP. In the absence of ATP *K*_D_ values were too high to be determined for Mg^2+^ or Ca^2+^ as no detectable binding was observed at a ssDNA concentration 10-fold higher than the *K*_D_ values we had obtained in the presence of ATP. A measurable *K*_D_ of 82.4 nM ± 13.2 (±standard deviation) was determined for Mn^2+^. This result with Mn^2+^ was very similar to Mg^2+^ in the presence of ATP but Ca^2+^ substantially destabilized the SsoRadA–ssDNA interaction. Destabilization could explain tolerance of SsoRadA-mediated strand exchange to ATPγS in the presence of Ca^2+^, although additional study is needed to determine if destabilization provides greater flexibility to filaments in the presence of Ca^2+^ or there is some other benefit conferred by ATP hydrolysis.

**Figure 8:**
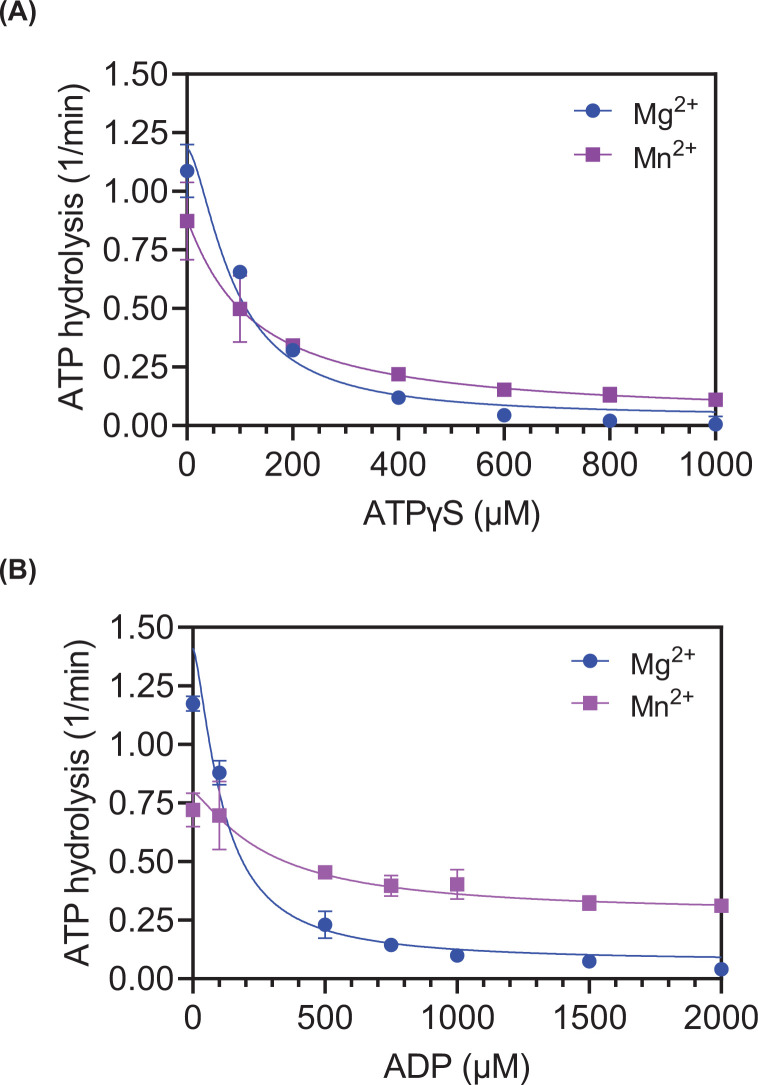
Inhibition of SsoRadA ATPase activity by ADP and ATPγS Fifteen-minute ATPase end points were assessed with increasing amounts of either ATPγS (shown in panel A) or ADP (shown in panel B). Graphed points are the average of three replicates and error bars are ± standard deviation. All reactions contained 500 μM ATP in addition to the indicated concentration of ATPγS or ADP. Lines shown are the nonlinear regression of inhibitor concentration versus response curves (GraphPad Prism 9). Blue circles represent reactions with Mg^2+^ and purple squares are those with Mn^2+^ and all error bars are standard deviation.

We next determined the IC50s for both ATPγS and ADP in the presence of Mg^2+^ or Mn^2+^, using the thin layer ATPase assay (Figure 8). The IC50 of ATPγS for SsoRadA with Mg^2+^ was 87.8 and 121 μM for Mn^2+^. As shown in Figure 8B, we obtained IC50 values of ADP for SsoRadA with Mg^2+^ of 112.4 and 282 μM with Mn^2+^. The manganese cation provides SsoRadA greater protection from inhibition by either ADP or ATPγS. This inhibition effect is greater for ADP than ATPγS. These results may in part explain what is observed in the strand invasion assay (Figure 3) where higher yields were obtained for assays containing Mn^2+^ relative to those with Mg^2+^, which are reduced by the addition of the ATP regeneration system to the assay.

## Discussion

In this work, we show the impact of varying divalent metal cofactors for a crenarchaeal RadA protein. We found that substituting Mg^2+^ with Mn^2+^ results in ATPase activity inhibition (Figure 2). Mn^2+^ utilization reduces the stability of one or more SsoRadA ADP-bound states but has little effect on the ATP bound states (Figure 8). Mn^2+^ also resulted in a substantial improvement in SsoRadA-mediated strand invasion activity (Figure 3). Taken together, these data suggest that Mn^2+^ enhances strand invasion by abrogating the ADP bound state of SsoRadA, a notion supported by the finding that strand invasion with Mg^2+^ is enhanced by ADP depletion (via the ATP-regeneration system) but the use of Mn^2+^ does not show similar increases. Interestingly, the Mn^2+^ effect does not lead to strand exchange enhancement, since both joint molecule and nicked circular product formation are reduced in the presence of this cofactor (Figure 5). Further structural analysis will be necessary to determine how Mn^2+^ gives rise to these seemingly opposite effects.

The effect of Ca^2+^ is much more dramatic than Mn^2+^ on SsoRadA ATPase activity, resulting in an almost complete abrogation of hydrolysis (Figure 2). Ca^2+^ destabilizes the association between free SsoRadA and ATP, and between SsoRadA-ATP and ssDNA. Use of Ca^2+^ in the strand exchange assay leads to a reduction of strand exchange activity, though with a milder effect than that observed for ATP hydrolysis (Figure 5), suggesting that Ca^2+^ may abrogate the need for ATP hydrolysis during strand exchange. Alteration of ATP hydrolysis through use of ATPγS with Ca^2+^ results in inhibition resistance (Figure 7), suggesting that Ca^2+^ substitution obviates a requirement for ATP hydrolysis through potential destabilization of the ATP-bound nucleoprotein filament.

Examination of the Walker box GKT ATPase mutants with different divalent cofactors gives additional insight. Strand invasion mediated by the K120R mutant is not affected by reduction of ADP by inclusion of the ATP regeneration system, but this activity is dramatically enhanced in the same conditions for the K120A mutant (Figure 4). Reducing available ADP has only a mild effect on K120R-mediated strand exchange but almost completely abrogates this activity for K120A with Mg^2+^ (Figure 6). Together, these results indicate that K120R-mediated processes are more resistant to the presence of ADP, suggesting either the ability to utilize ADP and ATP interchangeably for strand invasion/exchange or a reduction in ADP binding. K120A-mediated processes are instead dramatically affected by ADP depletion, indicating a higher affinity for ADP, which could imply an increased effect of this cofactor on the structure and/or binding ability of the mutant protein.

Previous characterization of crenarchaeal RadA proteins has been primarily focused on nucleoprotein filament structure and interactions with ssDNA, although ATPase activity and *in vitro* recombinase activity using Mg^2+^ as a cofactor have been examined [6,8,17,21,62,63]. Among the euryarchaea, most work has been with the recombinase proteins derived from *Pyrococcus furiosus* and *M. voltae*. Komori et al. determined that the C-terminal core domain of the *P. furiosus* protein (which is conserved across all archaeal recombinases) is sufficient for strand exchange [64]. Later efforts with this protein have largely focused on humanized versions used to test the interactions of small molecules and BRCA2 with human RAD51 [65–68]. When altered with a humanized ATP binding domain, *P. furiosus* RadA was found to exhibit a higher affinity for ADP than for ATP [69]. This outcome differs from our results with the SsoRadA protein, where ATPγS is a more effective inhibitor of SsoRadA ATPase activity than ADP (Figure 8). This incongruity could indicate a variance in cofactor affinity between the two proteins or could be the result of differences between stabilities of other ADP-bound states since the humanized *P. furiosus* RadA protein was evaluated in the absence of ssDNA [69]. Work with the *M. voltae* RadA protein has primarily focused on the effect of K^+^ and Ca^2+^ on activity and associated structure alterations. K^+^ stimulates both ATPase activity and strand exchange; at high concentrations K^+^ eliminates RadA ATPase activity dependence on ssDNA as well as the need for ATP hydrolysis for strand exchange [70,71]. Similarly, Ca^2+^ also eliminates the need for ATP hydrolysis in *M. voltae* RadA recombinase-mediated strand exchange [25]. The underlying cause of these effects relate to a second cation binding location within the ATPase site which allows binding of two divalent cations or a single divalent cation and two K^+^ ions [25,58,71]. This second site can bind Mg^2+^ but is thought to preferentially bind Ca^2+^ [25]. Salt bridges are formed between the cations, Asp-302 of the recombinase, and the phosphates of ATP, resulting in helical geometry changes and active filament formation [70]. Alignment of the *M. voltae* and *S. solfataricus* RadA proteins [21] reveals that Asp-302 corresponds to Asp-301 in the crenarchaeal protein, suggesting the possibility that the *S. solfataricus* RadA could also bind a second cation (such as Ca^2+^ or Mn^2+^) along with Mg^2+^.

Bacterial RecA proteins have been identified and characterized from many different species with the *E. coli* version most thoroughly examined. Both ATP hydrolysis and RecA-mediated strand exchange are dependent on Mg^2+^ and substitution with Ca^2+^ does not show the stimulatory effects observed for *M. voltae* RadA [25,47]. However, like the *M. voltae* recombinase, *E. coli* RecA exhibits salt-induced DNA-independent ATPase activity which results in the formation of an active nucleoprotein filament structure [70–73]. When compared to the *S. solfataricus* RadA protein, *E. coli* RecA activity with Ca^2+^ is similar and use of this cofactor reduces the rate of ATP hydrolysis (Figure 2) [47]. More recent studies of RecA ATPase activity determined that hydrolysis destabilizes the short strand exchange regions formed during homology search, resulting in increased kinetics for large strand exchange product formation [74]. Our observations of the effect of Ca^2+^ on invasion product stability and reduced ATPase activity for wild-type SsoRadA suggests that Ca^2+^ may reduce the rate of homology checking leading to persistence of D-loops (Figure 3). We did not, however, find a reduced rate of strand exchange when Ca^2+^ was used; this is distinct from results with *E. coli* RecA when ATPγS was used as a method to slow the homology check [74].

The effect of Ca^2+^ with human RAD51 has some similarity to that of Mn^2+^ with SsoRadA. For the human recombinase, Ca^2+^ has been shown to enhance strand invasion and the formation of strand exchange products [34,75]. This effect is believed to occur through a reduction of the amount of RAD51 protein bound to ADP by decreased ATPase activity. Our observations that Mn^2+^ enhances SsoRadA strand invasion activity, reduces the SsoRadA ATPase activity, and reduces the ability of ADP to inhibit SsoRadA could be considered comparable (Figures 2, 3, and 8). Additionally, Ca^2+^ has been found to improve the organization of the human RAD51 filament and increase its ability to displace RPA from ssDNA [57,76]. Further investigation will be needed to determine whether Mn^2+^ has a similar effect on SsoRadA filament organization and SSB displacement. Non-canonical cations other than Mg^2+^ alter the function of RecA family recombinases in all domains of life. For the SsoRadA protein and these alternative cations, ATPase activity and strand exchange are reduced while strand invasion is increased, which is distinct from the behavior of the *M. voltae* RadA protein. There is also a distinct difference when compared with *E. coli* RecA, except for reduced ATPase activity with alternate cations. Overall, the SsoRadA recombinase shows the most similarity to human RAD51 with Ca^2+^ for both strand invasion and exchange. This may be due to the similarities between DNA metabolism in archaea and eukaryotes. The effect of Mn^2+^ on the *S. solfataricus* RadA protein could result from the minerals present in the unusual environmental niche inhabited by this archaeon. While the overall effects of the non-canonical cations with SsoRadA examined in this work do reveal some similarities to previously investigated recombinases, the extent of activity alterations and preferred cofactor usage is unique to the *S. solfataricus* protein.

## Data Availability

All supporting data are included within the main article.

## Abbreviations

HR: homologous recombination
TLC: thin layer chromatography
*Tm*LDH: *Thermotoga maritima* lactate dehydrogenase
*Tm*PK: *Thermotoga maritima* pyruvate kinase
